# Predicting 1-Year Mortality after Hip Fracture Surgery: An Evaluation of Multiple Machine Learning Approaches

**DOI:** 10.3390/jpm11080727

**Published:** 2021-07-27

**Authors:** Maximilian Peter Forssten, Gary Alan Bass, Ahmad Mohammad Ismail, Shahin Mohseni, Yang Cao

**Affiliations:** 1 Department of Orthopedic Surgery, Orebro University Hospital, 701 85 Orebro, Sweden; maximilian.forssten@oru.se (M.P.F.); ahmad.mohammmad-ismail@oru.se (A.M.I.); 2 School of Medical Sciences, Orebro University, 702 81 Orebro, Sweden; garybassmd@gmail.com (G.A.B.); shahin.mohseni@oru.se (S.M.); 3 Division of Traumatology, Emergency Surgery and Surgical Critical Care, University of Pennsylvania, Philadelphia, PA 19104, USA; 4 Division of Trauma and Emergency Surgery, Department of Surgery, Orebro University Hospital, 701 85 Orebro, Sweden; 5 Clinical Epidemiology and Biostatistics, School of Medical Sciences, Orebro University, 702 81 Orebro, Sweden

**Keywords:** hip fracture, postoperative mortality, prediction, variable importance, machine learning, logistic regression

## Abstract

Postoperative death within 1 year following hip fracture surgery is reported to be up to 27%. In the current study, we benchmarked the predictive precision and accuracy of the algorithms support vector machine (SVM), naïve Bayes classifier (NB), and random forest classifier (RF) against logistic regression (LR) in predicting 1-year postoperative mortality in hip fracture patients as well as assessed the relative importance of the variables included in the LR model. All adult patients who underwent primary emergency hip fracture surgery in Sweden, between 1 January 2008 and 31 December 2017 were included in the study. Patients with pathological fractures and non-operatively managed hip fractures, as well as those who died within 30 days after surgery, were excluded from the analysis. A LR model with an elastic net regularization were fitted and compared to NB, SVM, and RF. The relative importance of the variables in the LR model was then evaluated using the permutation importance. The LR model including all the variables demonstrated an acceptable predictive ability on both the training and test datasets for predicting one-year postoperative mortality (Area under the curve (AUC) = 0.74 and 0.74 respectively). NB, SVM, and RF tended to over-predict the mortality, particularly NB and SVM algorithms. In contrast, LR only over-predicted mortality when the predicted probability of mortality was larger than 0.7. The LR algorithm outperformed the other three algorithms in predicting 1-year postoperative mortality in hip fracture patients. The most important predictors of 1-year mortality were the presence of a metastatic carcinoma, American Society of Anesthesiologists(ASA) classification, sex, Charlson Comorbidity Index (CCI) ≤ 4, age, dementia, congestive heart failure, hypertension, surgery using pins/screws, and chronic kidney disease.

## 1. Introduction

Some patient-level evidence exists associating comorbidity burden with short-term survival after hip fracture surgery [1]. Postoperative death within 1 year following hip fracture surgery is also relatively common, with reported estimates up to 27% [2,3]. Thus, given the per annum denominator of approximately 18,000 hip fractures, this disease accrues excess morbidity, mortality and an annual direct cost of approximately 1.5 billion SEK (175 million USD/147 million EUR) to the Swedish healthcare system [4,5,6]. Costs are predicted to increase with an ageing population [7].

There is a potential utility in outcome prediction, particularly in better matching perioperative resources to this population’s needs. The logistic regression (LR) method of prediction is well-known to researchers and surgeons, and our recent work demonstrated that convolutional neural networks do not outperform LR in predicting 30-day postoperative mortality in hip fracture patients [8]. There are, however, other viable alternatives that should be explored. Support vector machines (SVM), for example, have been shown to require fewer variables than LR to achieve a lower, or equivalent, misclassification rate [9]. A naïve Bayes classifier (NB) offers speed advantages when fitting the model [10], while random forest classifiers (RF) often outperform LR in feature-rich datasets [11]. In the current study, we benchmarked the predictive precision and accuracy of SVM, NB, and RF against LR in predicting 1-year postoperative mortality in hip fracture patients as well as compared the relative importance of the variables included in the LR model.

## 2. Materials and Methods

### 2.1. Study Population

The principles of the Transparent reporting of a multivariable prediction model for individual prognosis or diagnosis (TRIPOD) guidelines were adhered to while conducting this study (Table S1). We used the high-fidelity Swedish National Quality Register for Hip Fracture Patients, RIKSHOFT, to identify a study population composed of all adult patients (18 years or older) who underwent primary emergency hip fracture surgery in Sweden, between 1 January 2008 and 31 December 2017 [12]. Patients with pathological fractures and non-operatively managed hip fractures, as well as those who died within 30 days after surgery, were excluded from the current analysis. Studying the association between the variables included in the prediction models and mortality in the first year, conditional on patients surviving 30 days or more after surgery, allows for the evaluation of the long-term association between the potential predictors and mortality, in isolation. This may reduce the effect of short-term mortality due to surgical and anesthesiologic complications as well as the higher degree of advanced directives present in the study population compared to the general population. 

The selected patients were cross referenced with the Swedish National Patient and Cause of Death registers maintained by the Swedish National Board of Health and Welfare to retrieve comorbidity and mortality data. Variables used in the prediction model include survival status one year after surgery, age, sex, American Society of Anesthesiologists (ASA) classification, age-adjusted Charlson Comorbidity Index (CCI) [13], Revised Cardiac Risk Index (RCRI) [14], concurrent comorbidities (arrhythmia, hypertension, myocardial infarction, congestive heart failure, cerebrovascular disease, chronic obstructive pulmonary disease, connective tissue disease, dementia, diabetes mellitus, liver disease, hemiplegia, chronic kidney disease, local tumor, metastatic carcinoma, peptic ulcer disease), type of fracture, and surgical procedure performed.

### 2.2. Descriptive Analysis

Continuous variables were presented as a mean ± standard deviation (SD), and the ordered and nominal variables were presented as a count and percentage. The Pearson’s chi-squared test was used to test the statistical significance of differences between groups for categorical variables, while the Student’s t-test and Mann–Whitney U test were used for continuous variables [15,16,17]. Missing values were imputed using the chained method with the nearest neighbor algorithm [18,19]. Two-tailed *p*-values < 0.05 were considered statistically significant.

### 2.3. Predictive Models

A LR model with an elastic net regularization (the final ratio of L1 penalty: L2 penalty = 0.5:0.5 based on the random search) was used in the current study [20,21]. Two LR models were constructed, one including all the included variables and the other including the 10 most important variables. The relative importance of the variables was evaluated using the permutation importance (PI) [22]. PI was measured by looking at how much a predefined performance metric (in the current study Youden’s index, i.e., the sum of sensitivity and specificity minus 1, was used) is impacted by missing specific variable data [23]. To mask the information of a variable during evaluation, instead of removing the variable from the dataset, the PI method replaces it with random noise from other participants by shuffling the values of the variable. The relative importance of a variable was then calculated as the decrease in Youden’s index of the variable relative to the range of the decreases of all the variables [22]. LR was also compared with three other commonly used supervised classification algorithms, i.e., NB, SVM, and RF, and presented using a calibration plot. NB is a probabilistic classifier based on applying Bayes’ theorem with strong (naïve) independence assumptions between the variables. It can be trained very efficiently in a supervised learning setting. SVM is an algorithm aiming to find a hyperplane in an N-dimensional space (N is the number of variables) that may distinctly classify the data points. RF is an ensemble learning method for classification, regression and other tasks that operates by constructing a multitude of decision trees at training time. For classification tasks, the output of RF is the class selected by most trees [24,25,26,27].

### 2.4. Data Normalization and Model Construction

Multinomial variables were converted into multiple binary variables before entering the models. Ordinal and continuous variables were normalized using the min–max transformation to have values between 0 and 1 [28]. Eighty percent of the randomly selected patients were used as a training dataset to train the LR models. During the model training stage, K-fold cross-validation was used [29]. The training dataset was randomly split into five equal partitions, which instantiated five identical model building and validation processes. The LR models were built on four partitions while the predictive ability was evaluated using the remaining partition. The predictive ability of the models and importance of the variables based on the training dataset were calculated as the average over the five validations. The remaining 20% of the patients were used as a test dataset to provide an unbiased evaluation of the final models’ fit on the training dataset.

Because tuning of hyperparameters, which define the model architecture, is an important issue for model optimization, during the model training, we used the recommended random search method to find the optimal hyperparameters for the compared machine learning algorithms, including the penalty parameters for the LR models. The method searches the hyperparameters based on presumptive distributions (both Gaussian distribution and uniform distribution were implemented in the current study) from which values may be randomly sampled. The method is able to find the optimized models within a small fraction of the computation time that are as good as or better than those based on the exhaustive grid search method [30].

### 2.5. Metrics of Predictive Ability

Given that our prediction task was a binary classification question, i.e., whether the patient would die or survive, we used the threshold-dependent metrics, including overall accuracy, sensitivity, specificity, to evaluate the performance of the LR models [31]. In the current study, we reported the accuracy, specificity, and sensitivity at the threshold that maximized the Youden index (or sensitivity + specificity −1) [32]. The receiver operating characteristic (ROC) curve and the area under the ROC curve (AUC) with 95% confidence interval (CI) were also reported based on the model-predicted probabilities. The boundaries for acceptable, good, and great predictive models were defined as an AUC value greater than 0.7, 0.8, and 0.9, respectively [33].

The descriptive analysis was conducted in R 4.0.3 (R Foundation for Statistical Computing, Vienna, Austria, https://www.r-project.org (accessed on 9 May 2021)). The creation of the machine learning models and calculation of each model’s predictive ability along with the PI were performed in Python 3.7 (Python Software Foundation, https://www.python.org (accessed on 9 May 2021)) using the Scikit-learn 0.24 and ELI5 0.11.0 packages.

## 3. Results

### 3.1. Patient Demographics

We identified 124,707 traumatic hip fracture cases who survived beyond 30 days after their operation, of whom 21,045 (16.9%) died within the 1-year postoperative period. The patients who died were on average older and more often male. Patients who died tended to have a higher comorbidity burden (CCI ≥ 7: 31.2% vs. 14.3%, *p* < 0.001), were less fit for surgery based on their ASA classification (ASA ≥ 3: 74.9% vs. 53.0%), and had a higher cardiac risk (RCRI ≥ 2: 20.5% vs. 10.7%, *p* < 0.001) [1]. All comorbidities were more prevalent among patients who died except for hypertension and connective tissue disease (Table 1). 

### 3.2. Predictive Performance of the LR Models

**Figure 1:**
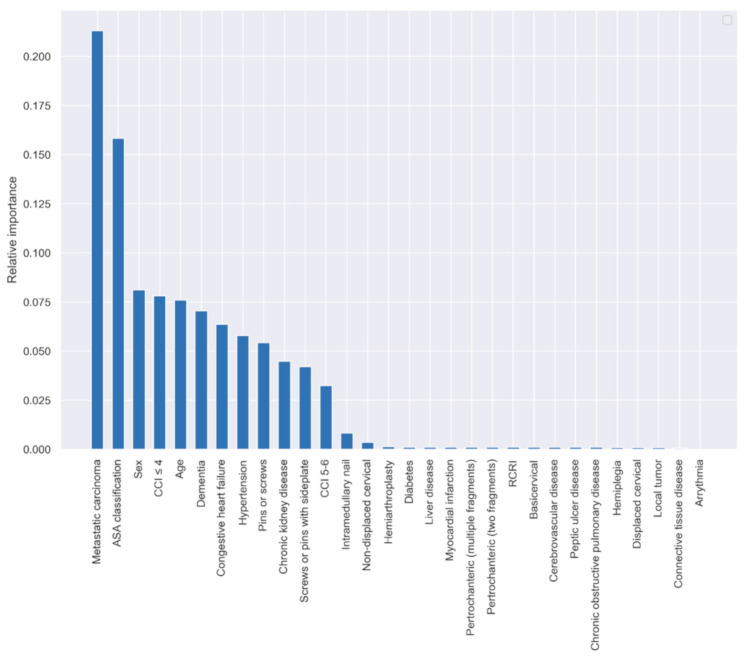
Relative variable importance in predicting one-year postoperative mortality based on the LR algorithm. ASA, American Society of Anesthesiologists; CCI, Charlson Comorbidity Index; RCRI, Revised Cardiac Risk Index.

The relative importance of the variables in predicting one-year postoperative mortality based on the LR algorithm and cross-validation using the training dataset is shown in Figure 1. The 10 most important variables were: metastatic carcinoma, ASA classification, sex, CCI ≤ 4, age, dementia, congestive heart failure, hypertension, surgery using pins/screws, and chronic kidney disease.

**Figure 2:**
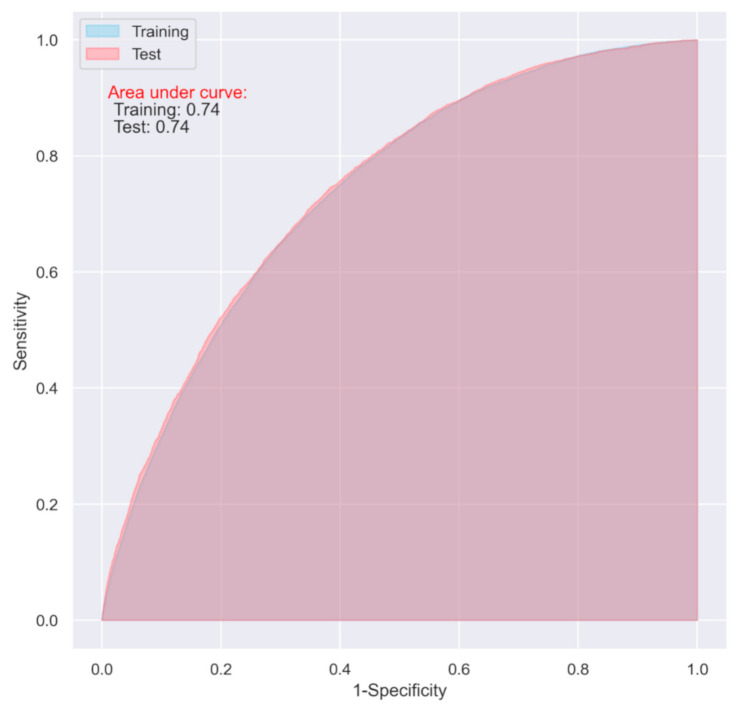
ROC of the LR model including all the variables with elastic net regularization.

**Figure 3:**
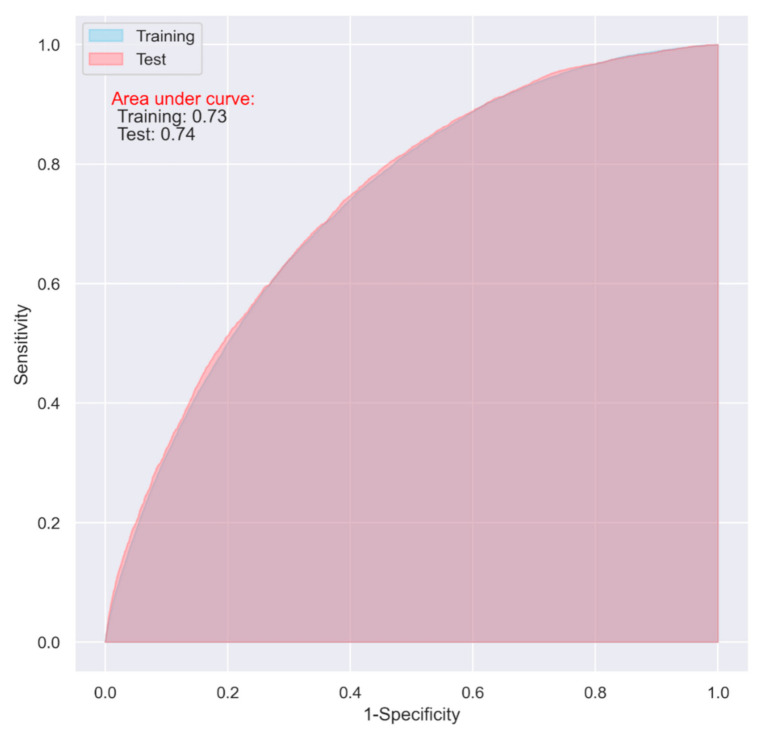
ROC of the LR model including only the top ten important variables with elastic net regularization.

The LR model including all the variables demonstrated an acceptable predictive ability on both the training and test datasets for predicting one-year postoperative mortality (AUC = 0.74 and 0.74 respectively) (Figure 2, Table 2). When including only the top ten most important variables, the model still achieved an acceptable predictive ability on both the training and test datasets (AUC = 0.73 and 0.74 respectively) (Figure 3, Table 2)

### 3.3. Comparison of LR with the Selected Machine Learning Algorithms

**Figure 4:**
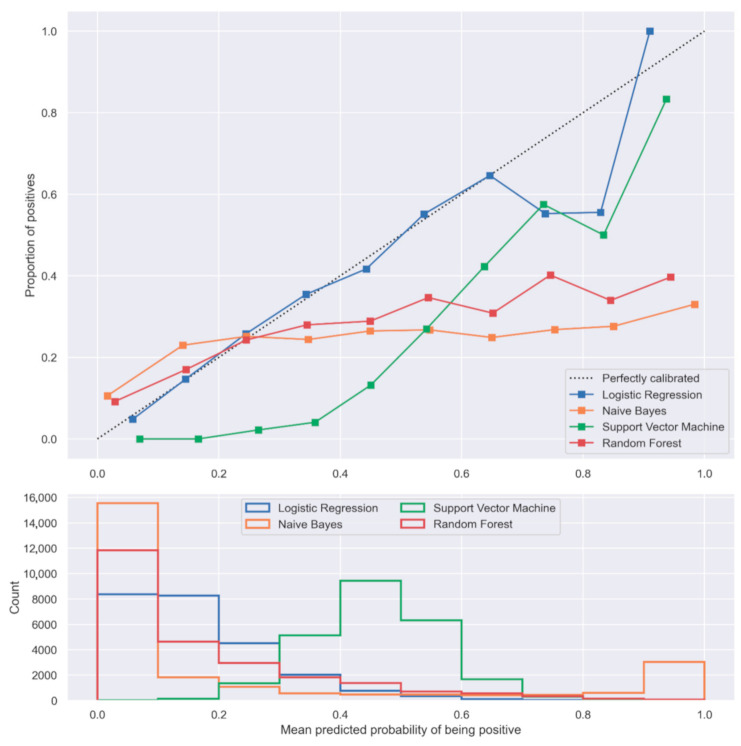
Calibration curves and histograms of the predicted probabilities of one-year postoperative mortality of the investigated machine learning algorithms.

In our dataset, the LR model showed superiority over the commonly used NB, SVM, and RF algorithms. In general, the three compared algorithms presented lower AUC, sensitivity, or specificity for both the training and test datasets (Table 2) and tended to over-predict the mortality overall as shown in Figure 4, where their calibration curves are all below the diagonal. The over-prediction was more prominent for NB and SVM algorithms, which significantly over-predicted mortality in the middle and upper end of the calibration curve (Figure 4). In contrast, LR only over-predicted mortality when the predicted probability of mortality was larger than 0.7 (Figure 4).

## 4. Discussion

A key benefit of precision medicine is the ability to predict the risk of mortality and determine when the marginal benefit of additional interventions is negligible. Prediction models have an important role to play, and as the models increase in sophistication the goal may be shifted from precision medicine tailored to *cohorts* to personalized medicine adapted for *individuals*. To date, this is the largest study investigating the performance of machine learning models in predicting 1-year postoperative mortality after hip fracture surgery beyond the admission/early 30-day mortality. We found that out of the tested algorithms, LR performed significantly better than the alternatives. While all others tended to overpredict mortality, the LR models only did so in higher risk patients. From a clinical perspective, this is not completely undesirable, as it efficiently flags high-risk patients in a population that is already at an elevated risk of mortality [1,3,34,35]. The LR model itself demonstrated an acceptable predictive ability, with an AUC of 0.74 in the test dataset using all variables and the top ten most important predictive variables [33].

As the name implies, NB assumes that the included features are conditionally independent; however, this is rarely the case in real datasets even if they can come close [36]. LR, on the other hand, splits the feature space linearly and is able to handle a certain degree of multicollinearity, particularly with the help of regularization [37]. NB has also been demonstrated to perform better on smaller datasets, while LR tends to gain the advantage as the training size increases [10,36]. This dataset is relatively large, and a degree of collinearity is undoubtedly present; the type of surgery selected for a hip fracture is in part dictated by the type of fracture and a patient’s age affects which comorbidities are present [38,39]. This, in combination with NB tending to have a higher bias than LR [10,36], likely all contributed to the LR models superior performance. This is important to note as large amounts of correlated data is the standard, rather than the exception, when working with data collected in the medical setting [40]. 

SVM makes use of the geometrical relationship between the features and outcome rather than the probabilistic model of LR [20,41]. This entails that SVM in most useful when working with unstructured and semi-structure data like text and images [42], while LR instead requires previously identified independent variables [20]. SVM also requires less features than LR to achieve an equivalent, or better, misclassification rate [9]. Conversely, the current dataset is feature rich and has clearly defined variables, which gives LR the overall advantage. In a broader context, this is also generally the case with medical big data [40]. The problems facing many researchers today is not a paucity in features, but rather an overabundance with a sometimes dubious accuracy and consistency [40].

The final machine learning algorithm that was investigated, RF, is an ensemble learning technique that combines the Bagging algorithm with the random subspace method while using decision trees as the classifier [43]. While not always the case [11], LR has previously been found to achieve a higher overall accuracy compared to RF [44]. LR also consistently outperforms RF in datasets with higher variance in their features [44]. As a whole, hip fracture patients are a heterogenous patient population; this is evident from both the distribution of risk scores, such as the ASA classification, RCRI, and CCI, in the current dataset as well as previous research [45]. Consequently, it is to be expected that LR surpasses RF in hip fracture patients. Nevertheless, in a larger medical context care should be taken to consider the specific population being studied as a higher degree of homogeneity may make RF a more viable option.

Of the variables analyzed, metastatic carcinoma, ASA classification, sex, CCI ≤ 4, age, dementia, congestive heart failure, hypertension, surgery using pins/screws, and chronic kidney disease were the most important for predicting mortality 1 year postoperatively. In contrast, a previous study from our group investigating 30-day postoperative mortality found that the most important predictors were age, hypertension, dementia, sex, ASA classification, RCRI, CCI, congestive heart failure, non-displaced cervical hip fractures, and cerebrovascular disease [8]. There was a significant overlap, with age, sex, ASA classification, CCI, dementia, congestive heart failure and hypertension being important for predicting both 30-day and 1-year mortality [8]. The increased importance of metastatic carcinoma and chronic kidney disease for predicting 1-year mortality is understandable as they are both chronic conditions with systemic, long-term effects; however, the remaining differences require further discussion.

Of particular note, the RCRI had previously demonstrated superiority over the CCI in predicting 30-day mortality [8]. However, while the CCI remained valuable for predicting 1-year mortality, the RCRI was significantly less useful. This is not altogether surprising as the RCRI was originally developed for predicting the 30-day risk of postoperative myocardial infarction, cardiac arrest, and mortality [46]. On the other hand, the CCI has been thoroughly validated for predicting 1-year mortality [13,47,48]. It is also worth noting that the CCI includes variables such as metastatic carcinoma, age, dementia, congestive heart failure, and chronic kidney disease in its calculation, all of which were variables that individually demonstrated the highest predictive ability for 1-year postoperative mortality in hip fracture patients. The fact that a CCI specifically ≤4 demonstrated a high importance is likely due to the fact this excludes or limits many of these previously mentioned comorbidities, which means this one variable contains a significant amount of information that is relevant to 1-year mortality. A patient with a CCI ≤ 4 cannot have a metastatic carcinoma since that would result in at least a CCI of 6. The patient must also either be old without any comorbidities, or relatively young with a few comorbidities, to stay at a CCI of 4; i.e., a patient in this category will undoubtably be healthier than the typical hip fracture patient [1,3,34,35]. 

Another significant change, compared to the prediction of 30-day mortality, was the inclusion of pins/screws rather than non-displaced cervical hip fractures as one of the variables with the highest predictive ability. To a certain extent these two variables can be considered interchangeable since the primary method used for fixating non-displaced cervical hip fractures is with pins/screws [38,39]. However, pins/screws are also at times used in the fixation of *displaced* femoral neck fractures, despite a consensus among orthopedic surgeons that hemiarthroplasty is the preferred surgical method for displaced femoral neck fractures in older patients [49,50,51]. This may be because of a belief that arthroplasty will result in a higher perioperative mortality in frailer patients, due to the increased stress caused by a longer period of time spent under general anesthesia with a more extensive surgical approach and intervention [50]. At least in patients with dementia, those who were operated on using pins/screws tended to have more comorbidities and were less fit for surgery [49]. An a priori preference for pin/screw fixation on the part of the operating surgeon may allow it to function as an indirect patient-level indicator of frailty, which could explain the variable’s increased importance for predicting 1-year mortality. 

In clinical practice several factors, such as age and comorbidities, have intuitively been associated with an increased mortality risk by physicians. Hip fractures predominantly affect older patients with several preexisting conditions [1,3,34,35,52]. As such, it is important to delineate other factors, which could be modifiable in the perioperative and postoperative period, that could aid in achieving better outcomes beyond the immediate postoperative period. For instance, while the RCRI does not excel at predicting 1-year mortality, it has been found to be predictive of, and associated with, worse short-term outcomes after emergency surgery [1,8,53,54]. This variable is readily available at the time of admission and for those with an increased cardiac risk, higher levels of care and cardioprotective medication such as beta-blockers are recommended by American Heart Association [55]. There is also evidence that patients with an elevated RCRI receive a greater survival benefit from beta-blocker therapy [35,56]. Another modifiable factor may be the type of surgery; observational studies indicate that hemiarthroplasty may be associated with lower mortality in patients with displaced femoral neck fractures [49,50,51]. 

This study makes use of the thoroughly validated Swedish National Quality Registry for Hip Fracture Patients, which is highly regarded for its high case coverage, encompassing between 80–90% of all hip fractures in Sweden [4,57]. The current dataset includes ten consecutive years of data from this database. Even so, the limitations of retrospective studies remain. The current analysis was limited to the data available in the registry so no anesthesiologic variables could be included in the prediction model, aside from the ASA classification. We also lacked data regarding more direct indicators of frailty, changes in functional status pre- and postoperatively as well as postoperative complications. Future studies investigating the role of frailty in predicting postoperative mortality as well as studying the predictors of postoperative complications are warranted.

## 5. Conclusions

LR outperformed the three other commonly use machine learning algorithms in predicting 1-year postoperative mortality in hip fracture patients. The most important predictors of 1-year mortality were the presence of a metastatic carcinoma, ASA classification, sex, CCI ≤ 4, age, dementia, congestive heart failure, hypertension, surgery using pins/screws, and chronic kidney disease. Further studies are required to determine the importance of frailty in predicting postoperative mortality in hip fracture patients.

## Figures and Tables

**Table 1:** Characteristics of the traumatic hip fracture patients in Sweden between 2008 and 2017.

Variable	Total (*n* = 124,707)	Alive (*n* = 103,662)	Dead (*n* = 21,045)	*p*-Value
Age, mean (SD)	81.55 (10.10)	80.75 (10.29)	85.46 (8.01)	<0.001
Sex, *n* (%)				<0.001
Female	86,379 (69.3)	73,166 (70.6)	13,213 (62.8)	
Male	38,314 (30.7)	30,483 (29.4)	7831 (37.2)	
Missing	14 (0.0)	13 (0.0)	1 (0.0)	
ASA classification, *n* (%)				<0.001
1	6356 (5.3)	6216 (6.1)	320 (1.6)	
2	46,507 (38.0)	41,626 (40.9)	4878 (23.6)	
3	60,857 (49.7)	48,278 (47.4)	12,579 (60.9)	
4	8459 (6.9)	5606 (5.5)	2853 (13.8)	
5	79 (0.1)	44 (0.1)	35 (0.2)	
CCI, *n* (%)				<0.001
≤4	57,634 (46.2)	52,281 (50.4)	5353 (25.4)	
5–6	46,733 (36.7)	36,604 (35.3)	9129 (43.4)	
≥7	21,340 (17.1)	14,777 (14.3)	6563 (31.2)	
RCRI, *n* (%)				<0.001
0	75,864 (60.8)	65,476 (63.2)	10,388 (49.4)	
1	33,476 (26.8)	27,135 (26.2)	6341 (30.1)	
2	11,284 (9.1)	8352 (8.1)	2932 (13.9)	
3	3245 (2.6)	2174 (2.1)	1071 (5.1)	
≥4	838 (0.7)	525 (0.5)	313 (1.5)	
Arrhythmia, *n* (%)	22,305 (17.9)	17,223 (16.6)	5082 (24.1)	<0.001
Hypertension, *n* (%)	47,990 (38.5)	39,849 (38.4)	8141 (38.7)	0.515
Myocardial infarction, *n* (%)	6789 (5.4)	5065 (4.9)	1724 (8.2)	<0.001
Congestive heart failure, *n* (%)	17,475 (14.0)	12,276 (11.8)	5199 (24.7)	<0.001
Cerebrovascular disease, *n* (%)	21,036 (16.9)	16,669 (16.1)	4367 (20.8)	<0.001
COPD, *n* (%)	13,933 (11.2)	11,094 (10.7)	2839 (13.5)	<0.001
Connective tissue disease, *n* (%)	6036 (4.8)	5017 (4.8)	1019 (4.8)	1.000
Dementia, *n* (%)	23,789 (19.1)	17,283 (16.7)	6506 (30.9)	<0.001
Diabetes mellitus, *n* (%)	18,166 (14.6)	14,588 (14.1)	3578 (17.0)	<0.001
Liver disease, *n* (%)	1232 (1.0)	985 (1.0)	247 (1.2)	0.003
Hemiplegia, *n* (%)	2715 (2.2)	2302 (2.2)	413 (2.0)	0.021
Chronic kidney disease, *n* (%)	5774 (4.6)	3920 (3.8)	1854 (8.8)	<0.001
Local tumor, *n* (%)	13,108 (10.5)	10,037 (9.7)	3071 (14.6)	<0.001
Metastatic carcinoma, *n* (%)	2498 (2.0)	1357 (1.3)	1141 (5.4)	<0.001
Peptic ulcer disease, *n* (%)	3918 (3.1)	3036 (2.9)	882 (4.2)	<0.001
Type of fracture, *n* (%)				<0.001
Non-displaced cervical (Garden 1–2)	16,840 (13.5)	14,138 (13.6)	2702 (12.8)	
Displaced cervical (Garden 3–4)	46,248 (37.1)	38,824 (37.5)	7424 (35.3)	
Basicervical	4126 (3.3)	3337 (3.2)	789 (3.7)	
Peritrochanteric (two fragments)	24,775 (19.9)	20,314 (19.6)	4461 (21.2)	
Peritrochanteric (multiple fragments)	22,487 (18.0)	18,502 (17.8)	3985 (18.9)	
Subtrochanteric	10,178 (8.2)	8501 (8.2)	1677 (8.0)	
Missing	53 (0.0)	46 (0.0)	7 (0.0)	
Type of surgery, *n* (%)				<0.001
Pins or screws	21,849 (17.5)	18,026 (17.4)	3823 (18.2)	
Screws or pins with sideplate	32,146 (25.8)	26,221 (25.3)	5925 (28.2)	
Intramedullary nail	29,496 (23.7)	24,461 (23.6)	5035 (23.9)	
Hemiarthroplasty	31,473 (25.2)	25,729 (24.8)	5744 (27.3)	
Total hip replacement	9676 (7.8)	9170 (8.8)	506 (2.4)	
Missing	67 (0.1)	55 (0.1)	12 (0.1)	

ASA, American Society of Anesthesiologists; CCI, Charlson Comorbidity Index; RCRI, Revised Cardiac Risk Index; COPD, Chronic obstructive pulmonary disease.

**Table 2:** Predictive ability of the investigated machine learning algorithms.

Model	Training			Test		
	Specificity	Sensitivity	AUC (95% CI)	Specificity	Sensitivity	AUC (95% CI)
LR	0.64	0.72	0.74 (0.73–0.74)	0.62	0.75	0.74 (0.74–0.75)
LR (including only the top ten variables)	0.65	0.73	0.73 (0.73–0.75)	0.61	0.74	0.74 (0.74–0.75)
NB	0.62	0.67	0.69 (0.69–0.70)	0.63	0.67	0.70 (0.69–0.70)
SVM	0.62	0.74	0.74 (0.73–0.74)	0.61	0.70	0.72 (0.72–0.73)
RF	0.57	0.73	0.71 (0.70–0.71)	0.66	0.67	0.72 (0.71–0.72)

## Data Availability

The ethical approval obtained for this study prevents the human data being shared publicly to protect patients’ privacy. Interested readers can contact Shahin Mohseni with their research plan to request access. This would be passed to Rikshöft who will decide whether they can access the data directly from the relevant Swedish authority.

## Supplementary Materials

The following are available online at https://www.mdpi.com/article/10.3390/jpm11080727/s1, Table S1: Transparent reporting of a multivariable prediction model for individual prognosis or diagnosis (TRIPOD) Checklist.
